# Role of prenatal imaging in the diagnosis and management of fetal facio-cervical masses

**DOI:** 10.1038/s41598-021-80976-4

**Published:** 2021-01-14

**Authors:** Weizeng Zheng, Shuangshuang Gai, Jiale Qin, Fei Qiu, Baohua Li, Yu Zou

**Affiliations:** 1 Department of Radiology, Women’s Hospital, Zhejiang University School of Medicine, Xueshi Rd No. 1, Hangzhou, Zhejiang People’s Republic of China; 2 Department of Ultrasound, Women’s Hospital, Zhejiang University School of Medicine, Xueshi Rd No. 1, Hangzhou, Zhejiang People’s Republic of China; 3 Department of Otolaryngology, Children’s Hospital, Zhejiang University School of Medicine, National Clinical Research Center for Child Health, Binsheng Rd No. 3333, Hangzhou, Zhejiang People’s Republic of China; 4 Department of Obstetrics, Women’s Hospital, Zhejiang University School of Medicine, Xueshi Rd No. 1, Hangzhou, Zhejiang People’s Republic of China

**Keywords:** Paediatric research, Ultrasonography, Magnetic resonance imaging

## Abstract

Congenital facio-cervical masses can be a developmental anomaly of cystic, solid, or vascular origin, and have an inseparable relationship with adverse prognosis. This retrospective cross-sectional study aimed at determining on the prenatal diagnosis of congenital facio-cervical masses, its management and outcome in a large tertiary referral center. We collected information on prenatal clinical data, pregnancy outcomes, survival information, and final diagnosis. Out of 130 cases of facio-cervical masses, a total of 119 cases of lymphatic malformations (LMs), 2 cases of teratoma, 2 cases of thyroglossal duct cyst, 4 cases of hemangioma, 1 case of congenital epulis, and 2 cases of dermoid cyst were reviewed. The accuracy of prenatal ultrasound was 93.85% (122/130). Observations of diameters using prenatal ultrasound revealed that the bigger the initial diameter is, the bigger the relative change during pregnancy. Magnetic resonance imaging (MRI) revealed that 2 cases of masses were associated with airway compression. In conclusion, ultrasound has a high overall diagnostic accuracy of fetal face and neck deformities. Prenatal US can enhance the management of ambulatory monitoring and classification. Furthermore, MRI provided a detailed assessment of fetal congenital malformations, as well as visualization of the trachea, presenting a multi-dimensional anatomical relationship.

## Introduction

Facio-cervical masses are frequent clinical findings in the pediatric population with only 55% of these lesions being congenital^1^. In fetuses, the common lesions include lymphatic malformations (LMs), dermoid cysts, cervical teratoma, thyroglossal duct cysts, hemangioma, goiter, and branchial cyst^2–4^. It is estimated that LMs occur in 1/1775 live births, and are more frequent in the facio-cervical masses (45–52%)^5^. Although it is rare in the fetal population, prenatal evaluation of fetal congenital facio-cervical masses is crucial during pregnancy^6, 7^. Congenital facio-cervical masses can be a developmental anomaly of cystic, solid, solid-cystic, or vascular origin, and have an inseparable relationship with intrauterine fetal death, structural anomalies, invasive procedures, cesarean delivery, dissatisfied cosmetic outcomes, or other adverse prognoses^8–10^. Therefore, the identification of a non-invasive and easily reproducible examination that could distinguish between fetal face and neck masses, is of great clinical importance.

Since head and neck anatomy is topographically complex, and the region is densely populated by vital nerves as well as vascular and lymphatic structures, perinatal management is a complex and challenging procedure^11, 12^. Therefore, an expert multidisciplinary team is the key to success, and it requires an understanding of the types of lesions and knowledge of the prenatal images that would best delineate the anatomic defect^2, 13^. Hence, accurate assessment depends on the proper determination of the involvement of the lesion.

In modern obstetrics, ultrasounds (US) are extensively used for prenatal diagnosis. Although these lesions have well-described imaging features, a careful assessment of specific risk factors using prenatal imaging is crucial. Advances in prenatal diagnosis and treatment technology have enhanced fetal management^14^. Therefore, craniofacial malformations associated with poor prognosis can be accurately treated with a good prognosis during the perinatal period^7, 14, 15^. In particular, magnetic resonance imaging (MRI), as an ancillary tool to US, is used for the diagnosis of fetal abnormalities. Despite the rapid increase in the number of cases that have been reported in literature, their prenatal and postnatal management is uncertain.

The aim of this study was to determine the diagnostic accuracy of prenatal US and MRI in the diagnosis of congenital facio-cervical masses. We evaluated the role of routine ultrasound for congenital facio-cervical masses. Moreover, imaging manifestations were retrospectively collected to determine the risk factors associated with inhibiting airway management, and to evaluate the potential relationship between fetal lesions and prognosis.

## Results

According to the inclusion and exclusion criteria, 143 cases of potential participants were included in the study. Simultaneously, their infants were followed for up to 1 year. Of the 143 fetal cases, 13 were lost during follow-up and were excluded from our study. Based on post-delivery follow-up (fetal autopsy, neonatal imaging, or operative and pathological findings), the perinatal features of the study population are shown in Table 1. For 130 cases of congenital facio-cervical masses, 52 mothers decided to continue the pregnancy and they were irrelevant to apparent chromosomal abnormalities.

**Table 1:** Perinatal features of the study population derived from 130 cases of fetal facial and cervical masses.

Characteristic	Value
**Maternal age** (**years**)	
Younger maternal age (< 35)	86.15 (112/130)
Advanced maternal age (> = 35)	13.75 (18/130)
**Lesions**^**a**^	
Lymphatic malformations	91.53 (119/130)
Teratoma	1.54 (2/130)
Thyroglossal Duct Cyst	1.54 (2/130)
Hemangioma	3.08 (4/130)
Congenital epulis	0.77 (1/130)
Dermoid cysts	1.54 (2/130)
**Delivery**	
Vagina	83.08 (108/130)
Caesarean section	16.92 (22/130)
**Pregnancy outcome**	
Live birth	40.00 (52/130)
Stillbirth	60.00 (78/130)
**Gestational age at delivery** (**weeks**)	
Live birth (weeks)	37.87 ± 3.02 (29–41)
Stillbirth (weeks)	15.38 ± 3.95 (11–29)

Data are given as % (n) or mean ± SD (range).

^a^The localization of the lesion for babies is diagnosed by postoperative pathologic results, postmortem, medical imaging.

**Figure 1:**
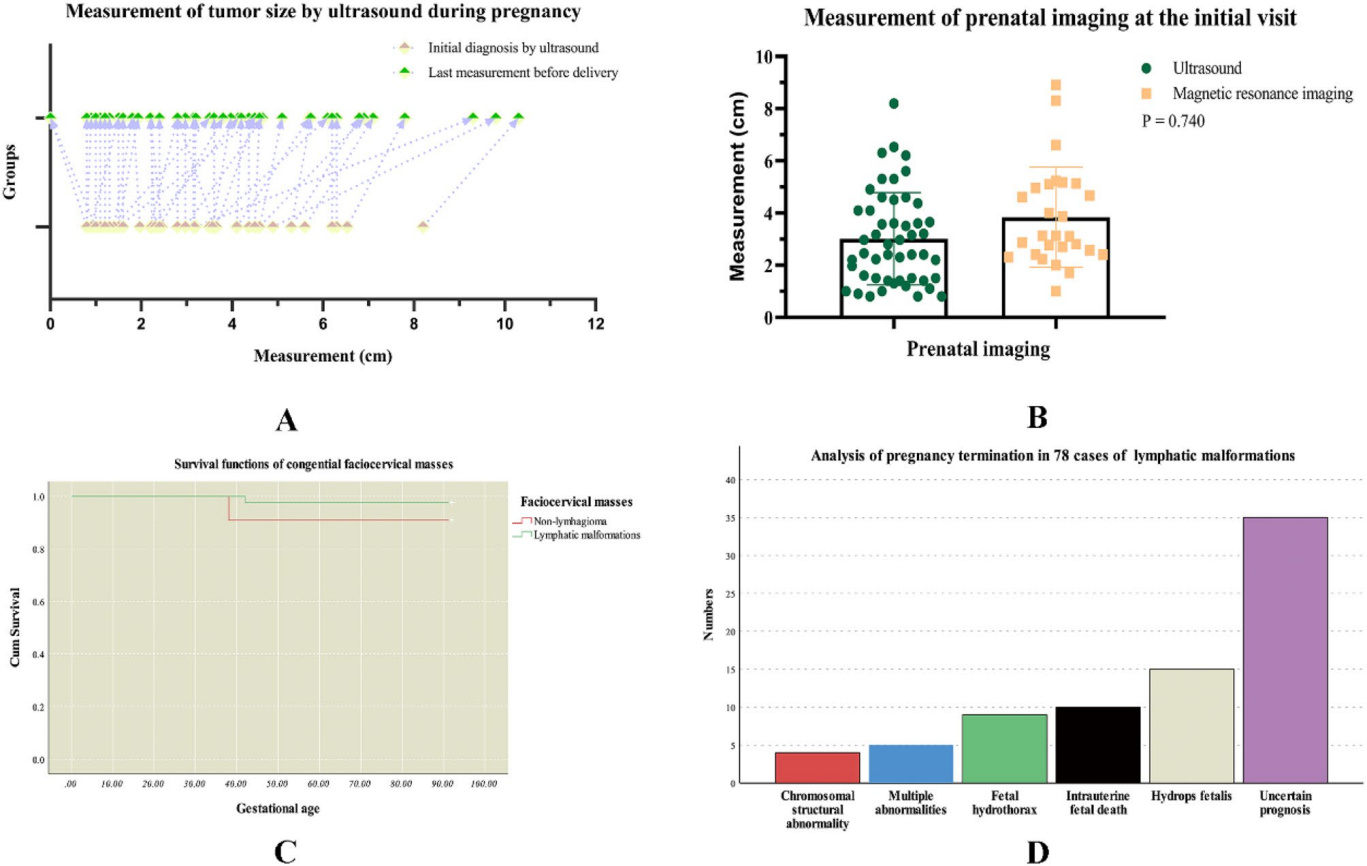
The figures show the survival information and prenatal imaging features of fetal facio-cervical masses.

For the 130 fetuses, the overall accuracy of prenatal US was 93.85% (122/130) while for 52 cases of liveborn infants, the accuracy of US was 84.62% (44/52). At the same time, MRI was performed in 28 cases among the 130 cases to obtain further details. The MRI accuracy was 92.86% (26/28). Compared to the prenatal US, MRI revealed 1 case of hemangioma and 1 case of lymphangioma. The mean GA at first visit was 26.88 ± 6.04 (range from 13 to 38) weeks for live births, while the mean GA at delivery was 37.87 ± 3.02 (rang 29–41) weeks. Prenatal ultrasound observations of diameters (Fig. 1A) revealed that the bigger the initial diameter is, the bigger the relative change during pregnancy. Moreover, the mean measurements between initial diagnosis and last measurement before delivery were statistically significant (*p* < 0.01). Furthermore, the statistical differences in the mean diameters between US and MRI cohorts were not significant (Fig. 1B). Based on these results, we firmly believe that the high diagnostic value and better dynamic range of prenatal US could be used to optimize prenatal care, and fetal MRI may have accessory diagnostic values during the perinatal period.

Ultrasonographic and clinical features of 52 live births cases are shown in Table 2. Moreover, among the surviving fetuses, 8 cases were accompanied by low Apgar score or poor perinatal prognosis (Table 3). This was attributed to premature infants (2 cases), airway obstruction (3 cases), and the big tumor size (3 cases). Of the 8 cases, invasive emergency treatment was performed on 3 newborns (cases 4, 7, 8). During prenatal imaging, ultrasound can assess fetal airway obstruction through indirect sign and dynamic imaging, such as the anatomic location of the tongue, fetal swallowing, polyhydramnios (case 4). MRI revealed that 2 cases of masses were associated with airway compression, thereby informing clinical management. Two fetuses (case 4, 7) were diagnosed with tracheal compression and/or throat compression during the perinatal period. However, in one newborn of oropharyngeal teratoma (case 8), neonatal death was encountered shortly after birth due to asphyxia. In one newborn with giant lymphangioma (case 6), the condition rapidly deteriorated after operative treatment, and respiratory failure with hypoxic-ischemic encephalopathy (HIE) was ultimately the cause of neonatal death. Moreover, for the 52 cases of liveborn infants, the survival curve plotted with survival information is shown in Fig. 1C. There was a superior survival rate of LMs (97.56%) compared to other cervical masses (90.91%). Based on these results, we can confirm that the prenatal imaging could indicate the high risks of infants depending on the imaging specific manifestations.

**Table 2:** Ultrasonographic and clinical features in 52 cases of the surviving fetuses.

Characteristic	Value
**Gender**	
Female	53.85 (28/52)
Male	46.15 (24/52)
**Delivery**	
Vagina	57.69 (30/52)
Caesarean section	42.31 (22/52)
Birth weight (g)	3251.92 ± 622.97 (1180.00–4410.00)
**Apgar score**	
1 min	9.56 ± 1.23 (5–10)
5 min	9.65 ± 1.10 (5–10)
**Localization of lesions**	
Posterior triangle	42.31 (22/52)
Anterior triangle	34.62 (18/52)
Middle neck/submental triangle	15.38 (8/52)
Oral cavity	7.69 (4/52)
**Ultrasonographic features**	
Septation	61.54 (32/52)
Blood flow	21.15 (11/52)
Solid-cystic	13.46 (7/52)
Turbid fluid	5.77 (3/52)
Polyhydramnios	17.31 (9/52)
Wall thickening	11.54 (6/52)
Gestational age at first visit (weeks)	26.88 ± 6.04 (13–38)
Systolic/diastolic (S/D) ratio	2.52 ± 0.64 (1.60–4.30)

Data are given as % (n) or mean ± SD (range).

**Table 3:** Clinical analysis of 8 cases of fetuses with poor perinatal prognosis.

Characteristic	Cases							
	1	2	3	4	5	6	7	8
Lesions	Hemangioma	Lymphangioma	Lymphangioma	Lymphangioma	Lymphangioma	Lymphangioma	Teratoma	Teratoma
Cystic and/or Solid	Solid-cystic	Cystic	Cystic	Solid-cystic	Cystic	Cystic	Solid-cystic	Solid-cystic
Mode of delivery	CS	VD	CS	CS	VD	CS	CS	VD
Localization of lesions	Anterior triangle	Anterior triangle	Posterior triangle	Anterior triangle	Posterior triangle	Posterior triangle	Anterior triangle	Oral cavity
Apgar scores at 1, 5 min	9–7	9–9	9–10	7–8	7–8	10–10	5–5	5–5
Gestational age at delivery	29	40	39	38	29	39	30	38
Size at delivery (cm)	3.0*3.1*1.6	11.8*10.3*7.3	8.1*9.7*3.0	4.4*8.0*4.8	1.5*0.9*1.3	11.5*12.5*6.2	7.6*5.6*8.3	5.7*6.0*7.0
Birth weight (g)	1180	3820	3750	2960	1610	3860	1380	2300
Gender	Male	Male	Male	Female	Male	Male	Female	Female
Prenatal diagnosis	Omission diagnosis	US + MRI	US	US + MRI	US	US + MRI	US + MRI	US
Airway obstruction on prenatal imaging	Omission diagnosis	No	No	US + MRI	No	No	MRI	No
Perinatal emergency treatment	Ventilator support	Ventilator support	No	EXIT	Ventilator support	No	Endotracheal intubation	Endotracheal intubation
Pregnancy outcome	live birth	live birth	live birth	live birth	live birth	Neonatal death	live birth	Neonatal death

*CS* cesarean section, *VD *vaginal delivery, *EXIT* *ex-utero* intrapartum treatment.

Due to the complexity and poor prognosis of cervical LMs, there was a higher rate of termination of pregnancy (TOP) (65.54%, 78/119) compared to the other cervical masses. During the early pregnancy and middle pregnancy stages, the mean GA of pregnancy termination was 15.38 ± 3.95 (rang 11–29) weeks. Etiological analysis of the TOP fetuses revealed some causative factors (Fig. 1D). Moreover, for the prenatal management of cervical LMs, fetal mortality was high.

## Discussion

Congenital conditions of facial and cervical structures that are associated with fetal growth, genetic diseases, fetal death, and upper airway obstruction, are associated with significant morbidity and may even be fatal^16, 17^. Rapid advances in imaging and instrumentation technology combined with superior knowledge of fetal pathophysiology, have led to the development of novel intrapartum interventions for most common fetal anomalies^18–20^. Furthermore, the assessment of facio-cervical lesions in utero can have significant importance in obstetric management since masses in this anatomical space may change rapidly and obstruct the airway. In the present study, we established that ultrasound has a high overall diagnostic accuracy for fetal face and neck deformities. Prenatal US could enhance ambulatory monitoring and classification, and may help clinicians improve their treatment modalities. Furthermore, prenatal MRI is an effective supplement for US and can be used to visualize the displacement and distortion of the fetal trachea, which is affected by the cervical huge masses.

**Figure 2:**
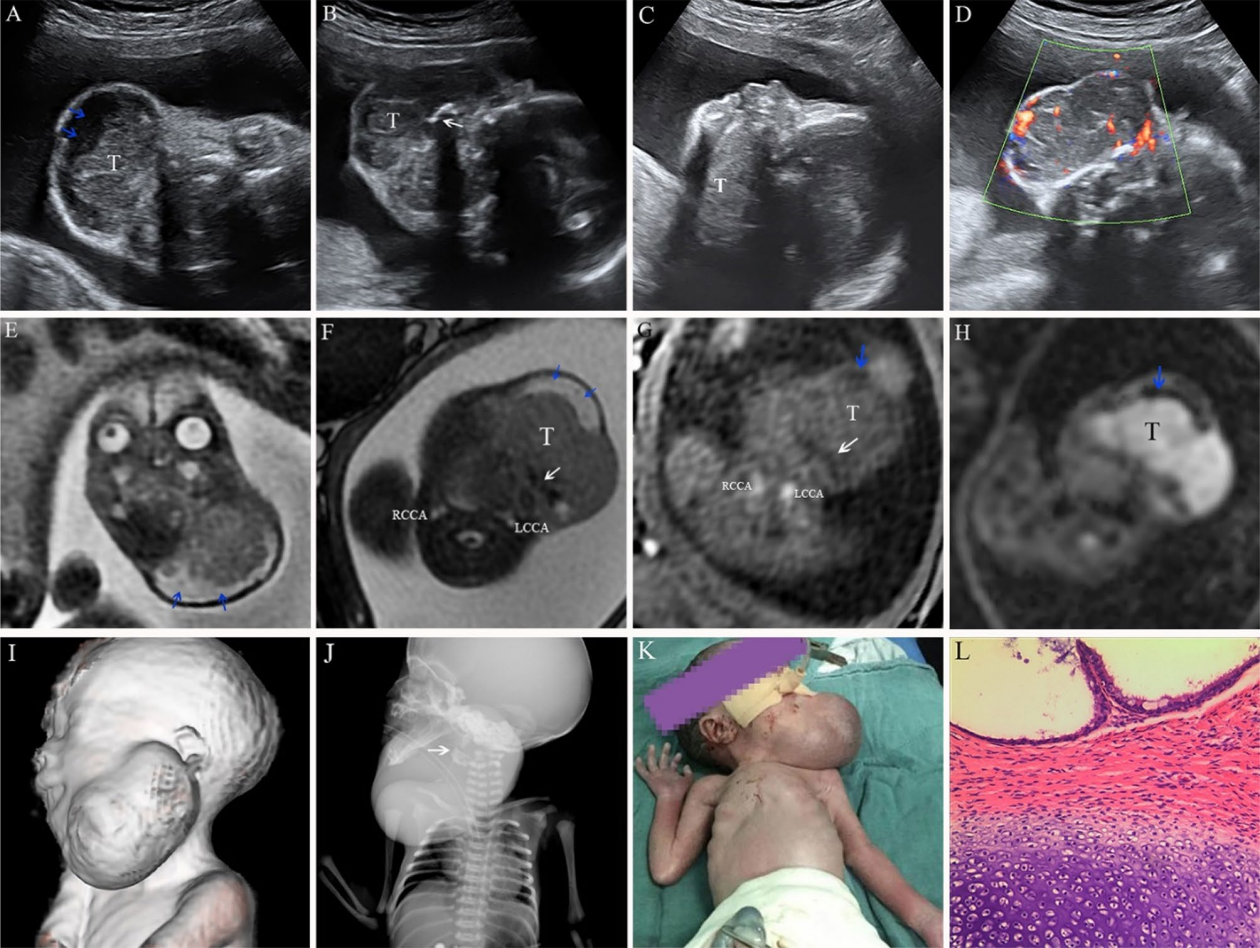
A submandibular teratoma at 29 weeks of gestational age. (**A–D**) Upon prenatal ultrasonic imaging, we found that the giant tumour (T) was with a slightly high intensity echo, high echo lesions with posterior acoustic shadowing at the central region (white arrow), capsule echo at marginal region (blue arrow). Color Doppler imaging showed rich internal blood flow. (**E–I**) On fetal MRI, the lesion was cystic-solid, calcified foci is striped low signal. Diffusion weighted imaging (DWI, b = 800) showed diffusion restriction in solid region (Fig H). Left and right internal carotid arteries were clearly visible (Fig F, Fig G). MR volume reconstruction (VR) image clearly showed the relationship and morphology of mass. (**J**) Neonatal radiography demonstrated huge high-density lesions with calcification (white arrow) under the jaw. (**K**) Image of the newborn after endotracheal intubation. (**L**) The pathological specimen was confirmed to be a mature cystic-solid teratoma (HE staining, × 100 magnification).

**Figure 3:**
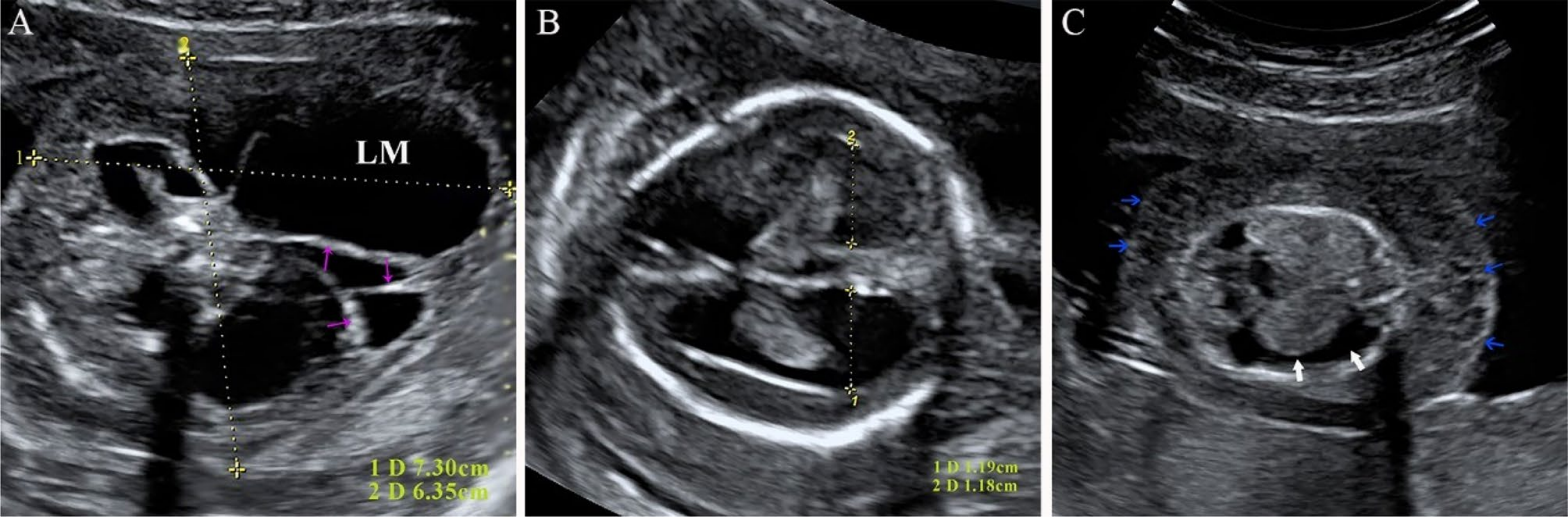
A lymphatic malformation (LM) at 17 weeks of gestational age. (**A**) Prenatal ultrasound exhibited a large anechoic lesion (7.3 × 6.35 cm) located at the back of the neck, and multiple intervals in the tumor can be identified. (**B**) The posterior horn of the right and left lateral ventricles were 1.18 cm and 1.19 cm, respectively. (**C**) Ultrasound of fetal chest showed a large pleural effusion (white arrow) and demonstrated severe fetal subcutaneous edema with low unevenness echo (blue arrow).

**Figure 4:**
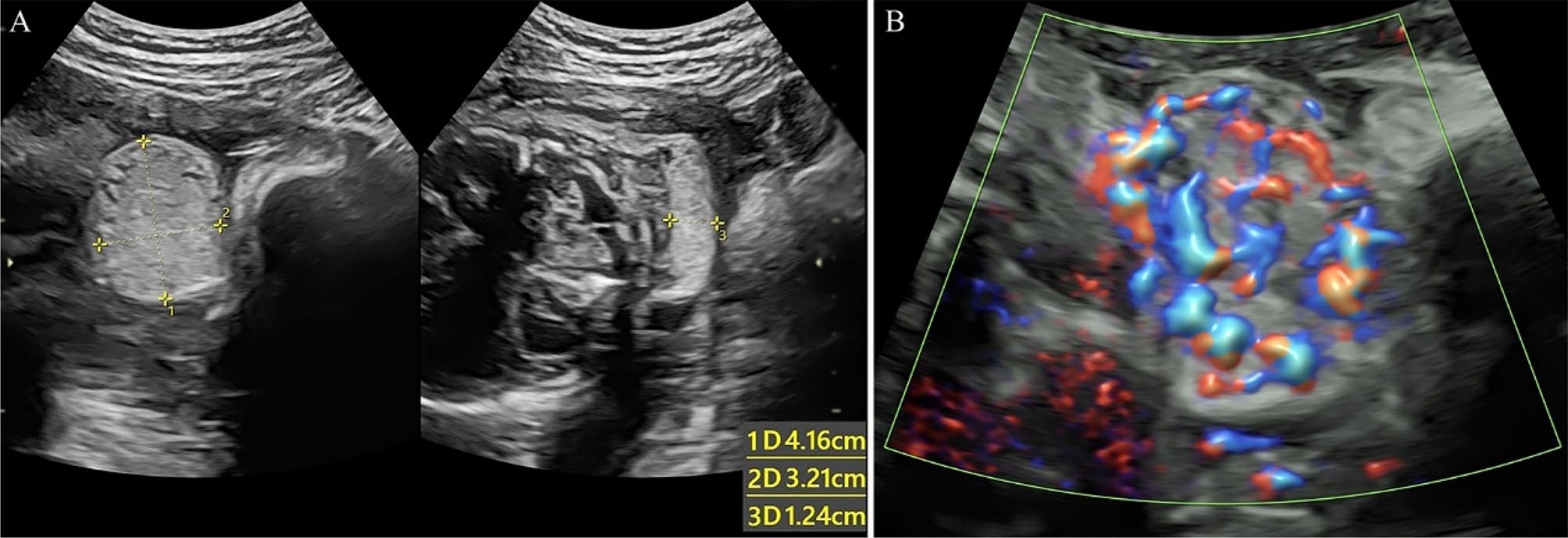
A hemangioma at 26 weeks of gestational age. (**A**) Prenatal ultrasound exhibited a hyperechogenic lesion (4.16 × 3.21 × 1.24 cm) located at the left of the neck. (**B**) Color Doppler ultrasound of blood flow showed rich blood flow signals inside the mass.

**Figure 5:**
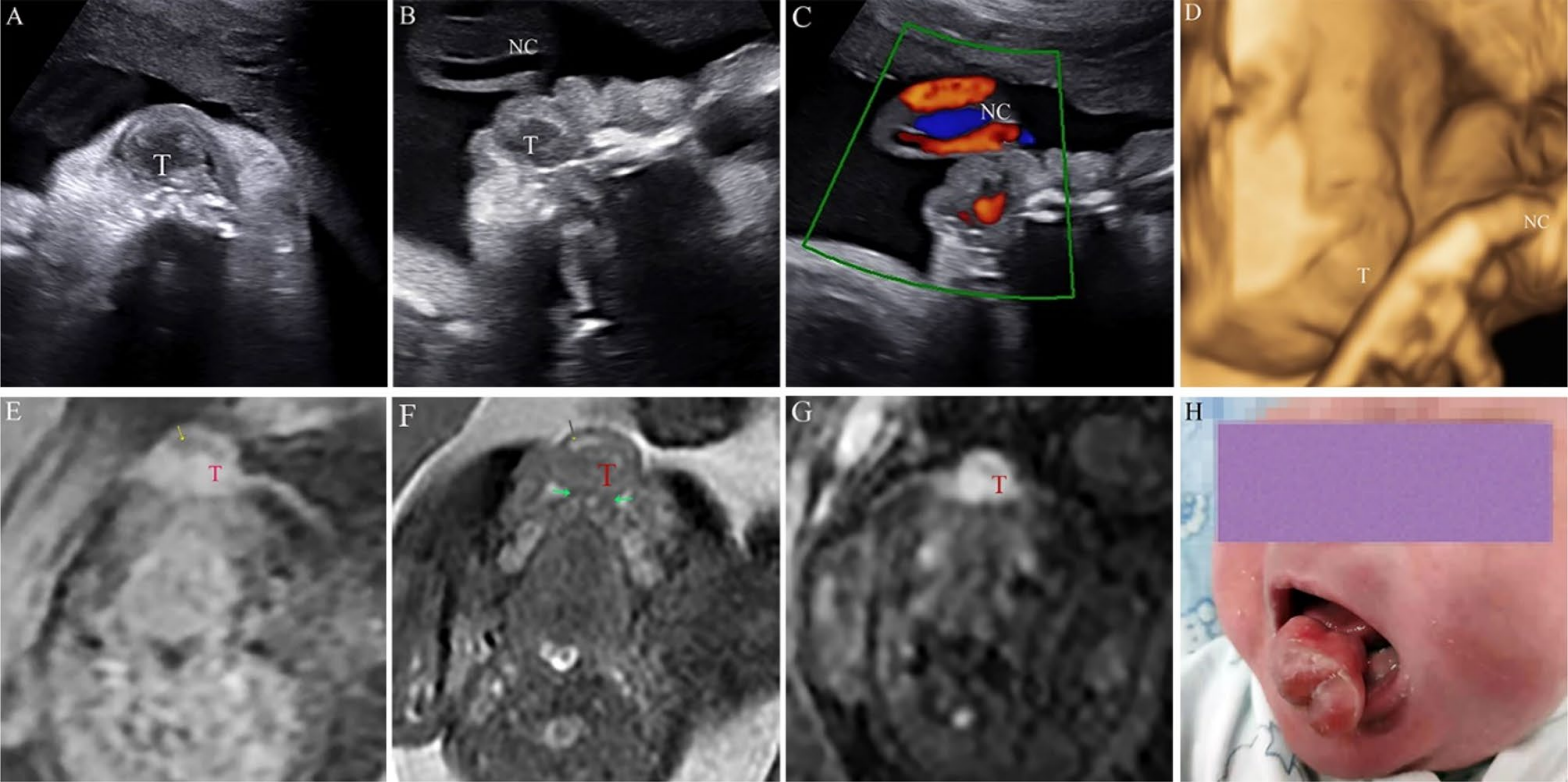
A congenital epulis at 34 weeks of gestational age. (**A–B**) On prenatal ultrasonic imaging, echography imaging revealed a low uneven echo mass (T) in the oral cavity that is lobulated with clear boundaries, an irregular shape. (**C**) Color Doppler ultrasound of blood flow showed abundant blood flow signals inside the mass, and nuchal cord (NC) was also shown. (**D**) Three-dimensional ultrasound of fetus, and was outward. (**E**) On T1WI, the lesion was with isointense signal and the peripheral part displayed a hypointense signal (yellow arrow). (**F**) On T2-weighted fat suppression image, the fat tissue was not detected while the mass was closely associated with the mandibular tooth bud (green arrow). (**G**) DWI image showed hyper-intensity in solid lesions of tumor tissues. (**H**) An image of the newborn.

In our current study, we collected 119 cases of LMs, 2 cases of teratoma, 2 cases of thyroglossal duct cyst, 4 cases of hemangioma, 1 case of congenital epulis, and 2 cases of dermoid cyst. The overall accuracy of prenatal US was 93.85% (122/130). The accuracy of MRI was 92.86% (26/28). Moreover, MRI revealed 1 case of hemangioma and 1 case of lymphangioma. An antepartum examination of the neck with a clear understanding of embryology and anatomy of the region will facilitate diagnosis. Defective embryologic development of LMs included embryonic sequestration of lymphatic rests, and the developmental failure of the juguloaxillary lymphatic sac to connect to venous system resulting in obstruction of lymphatic drainage^21, 22^. Moreover, Gedikbasi et al.^23^ using prenatal ultrasound, found that trisomy 21 is associated with non septated cystic hygroma while Turner syndrome is associated with septated cystic hygroma. Structurally, they may be characterized as microcystic, macrocystic, or combined lesions and these properties have significant clinical implications in treatment^24^. Congenital lesions are comprised of ectopic tissues arising from ectoderm or embryologic germ cells that may develop into the benign cyst (epidermoid or dermoid) or usually benign neoplasm (teratoma)^25^. According to our ultrasound features, for cervical teratoma (Fig. 2) and LMs (Fig. 3), cystic and solid tumor components as well as intratumoral septation could be reliably differentiated. Furthermore, thyroglossal duct cyst is a congenital cyst in the anterior midline of the neck that occurs due to a failure of the thyroglossal duct to involute completely during weeks 8–10 of gestation^26^. Therefore, US revealed an isolated cystic mass on the anterior midline of the neck, indicating a radiological diagnosis of thyroglossal duct cyst. Congenital hemangiomas are vascular lesions that are fully formed at birth, however, their aetiology is unknown^27, 28^. In Fig. 4, the color Doppler ultrasound of blood flow showed rich blood flow signals inside the hemangioma. Congenital epulis (Fig. 5) was extremely rare in the facial masses. Our results, along with other previous studies, show that the features of neck lesions such as size, invasion, septation, calcification, blood supply, cystic or/and solid lesion, turbid fluid, and so on were well evaluated on prenatal US (Table 2). The multi-modal fetal MRI was a useful adjunct for detecting components of the tumors, and would enhance confidence in the final diagnostic results.

In 52 live births cases, the localization of lesions were respectively at the posterior triangle (42.31%), anterior triangle (34.62%), middle neck or submental triangle (15.38%), and oral cavity (7.69%). Prenatal imaging was used to determine the disease process, location, origin, extent, and resectability of the lesions^13, 29^. Additionally, through regular follow‐up by prenatal ultrasound, we found that the bigger the initial diameter is, the bigger the relative change during pregnancy, and 2 cases were spontaneous. Moreover, the mean measurements between initial diagnosis and last measurement before delivery were statistically significant (*p* < 0.01). According to previous studies^30, 31^, a sudden increase in size may indicate intratumoral hemorrhage. Therefore, clinical management of its dynamic growth during middle or late gestation in pregnancy is important. Therefore, routine ultrasound screening in the face and neck during pregnancy is well established, and accurate management of a neck mass is critical.

Despite congenital facio-cervical masses being generally benign and developmental^32^, abnormalities may be seen in isolation or in association with central nervous system abnormalities, chromosomal abnormalities, intrauterine fetal death, and adverse prognosis^10, 33^. First-trimester cystic hygroma is a frequent finding in general obstetric screening programs. In this study, there was a higher rate of TOP of LMs compared to other cervical masses. Four cases of chromosomal structural abnormality, 5 cases of multiple abnormalities, 9 cases of fetal hydrothorax, 10 cases of intrauterine fetal death, 15 cases of hydrops fetalis (Fig. 3), and 35 cases of subjective uncertain prognosis were found. During the early pregnancy and middle pregnancy stages, the mean GA of pregnancy termination was 15.38 ± 3.95 weeks. However, we found that only one out of the 41 cases of the newborns with LMs died after operative treatment, due to respiratory failure and HIE. For the newborns with at least 1 year of follow-up, they had a superior survival rate of LMs (97.56%) compared to other cervical masses (90.91%). Malone et al.^34^ documented that LMs had the strongest prenatal association with aneuploidy, with a significantly worse outcome compared to simple increased nuchal translucency. However, most pregnancies with normal evaluation after the second trimester resulted in a healthy infant with a normal pediatric outcome. This was consistent with our retrospective findings as well. Consequently, it would seem highly warranted to categorize the management of congenital facio-cervical LMs during the perinatal period.

**Figure 6:**
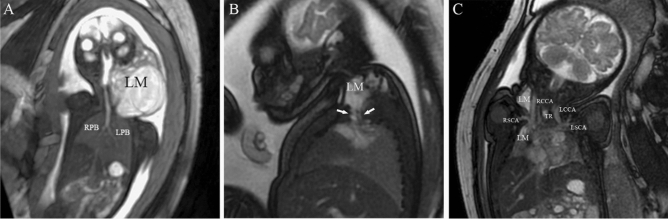
Two cases of lymphangioma (LM). (**A**) A case of LM at 26 weeks of gestational age (GA), the curved planar reformation of fast imaging employing steady-state acquisition (FIESTA) image showed high signal intensities of multilocular septum. The airway was filled with amniotic fluid showing a high signal, while the nasopharynx, laryngopharynx, trachea, and left primary bronchi (LPR) and right primary bronchi (RPR) were clearly displayed on level one. (**B**) For the fetus of LM at 35 weeks of GA, sagittal T2WI exhibited high signal cystic mass protrusions into the thoracic cavity from the right posterior triangle. (**C**) The coronal FIESTA image was same fetus as panel B, and showed a clear understanding of the relationship and anatomy of the region. RCCA = right common carotid artery, LCCA = left common carotid artery, RSCA = right subclavian artery, LSCA = left subclavian artery, TR = trachea.

The mass caused the disfigurement and destruction of adjacent tissues and structures. Fetuses with airway obstruction are at a high risk of hypoxic brain injury and death if delivered without proper preparation to guarantee prompt access to the airway in an efficient manner^35^. In this study, due to tracheolaryngeal obstruction, invasive emergency treatment was performed in 3 newborns with an Apgar score < 7 at 1 min (or < 8 at 5 min). The newborns involved were 2 cases of teratoma and 1 case of lymphangioma, respectively. Through dynamic imaging, an ultrasonologist assessed fetal airway obstructions such as glossocoma, dysphagia, and polyhydramnios (case 4). Once an abnormality was identified, three-dimensional ultrasound technology was used to describe the mass and swallowing and respiratory functions of the fetus in real-time. Furthermore, prenatal MRI was used to visualize tracheal compression and/or throat compression, and 2 cases of cervical huge masses were affected. Cervical masses were sometimes large and could extend to the floor of the mouth, involve the tongue (epignathus), or extend into the mediastinum. When amniotic fluid filled the fetal respiratory tract, coronal views of MRI were particularly important for visualizing the fetal airway; the nasopharynx, laryngopharynx, trachea, and bilateral primary bronchus. MRI provided a detailed assessment of fetal congenital malformations in our case series, as well as visualization of the trachea, presenting a multi-dimensional anatomical relationship of fetal malformations for the entire treatment team (Fig. 6). The *ex utero* intrapartum therapy (EXIT) procedure, as an important means of fetal surgery, is gradually used to secure the fetal airway before the complete delivery of the fetus^12^. Regrettably, a baby with Maxillofacial teratoma did not receive the evaluation of fetal MRI and the timely EXIT procedure. Eventually, treatment was abandoned by his parents due to severe dyspnea. Carol et al.^36^, David et al.^37^ and Timothy et al.^13^ reviewed the value of tracheoesophageal displacement index for EXIT-to-airway. A successful fetal surgery requires a multidisciplinary team, which is technically challenging as a treatment strategy for most hospitals^2, 38^. Therefore, evaluation of fetal airway enhances the appropriate management of fetal airway obstruction.

Potential limitations of our study should be noted. Our study was a single-center cross-sectional study of a retrospective nature, which may be biased. Perinatal management of congenital facio-cervical masses require special delivery strategies. Fetal and perinatal imaging is essential in the initial work-up, prenatal management, counseling, perinatal management, and follow-up of these fetuses. We hope that these interests will continue and expand.

## Conclusion

Detailed sonographic evaluation is of great significance in the determination of the prognosis of pregnancies that are complicated by congenital facio-cervical masses. The potential relationship between fetal lesions and viability is the basis for evaluating the suitability of perinatal management. Fetal MR imaging is a complementary imaging modality in the evaluation of fetuses with facial and neck lesions, which can be particularly useful when evaluating the effects of cervical masses within the airway.

## Materials and methods

### Ethic statement

We performed a retrospective cross-sectional study on fetuses referred to the Women’s Hospital, Zhejiang University School of Medicine, Hangzhou, China. Demographic information was collected from the medical records and PACS (picture archiving and communication system).

The personal data analysis, images of the newborn, and local pathological anatomy in this study were approved by the Institutional Review Board, Women’s Hospital, Zhejiang University School of Medicine (Approval Number: IRB-20200231-R). All methods were performed in accordance with the relevant guidelines and regulations of the National Health Commission of the People’s Republic of China. Written informed consent was obtained from the parents or legal guardians of the infants. All data were coded without identifying details and were used for research purposes only. Anonymity was promised, and neonatal privacy and protection were ensured.

### Patients

This study was conducted between January 2013 and August 2019. We included participants with fetal facio-cervical masses who had: (i) congenital tumors of the mouth, oral cavity, pharynx, and neck; ii. regular prenatal ultrasound examination, or with fetal MRI for prenatal diagnosis and iii. newborn with at least one year follow-up period. The exclusion was: (i) newborns lacking complete prenatal records or delivery records; ii. mothers referred to other agencies; iii. newborns who did not adhere to postnatal follow‐up. We observed the prenatal imaging features and collected the maternal–fetal clinical data. For stillbirths, we collected information on gestational age (GA) at birth, prenatal karyotype, the cause of termination of pregnancy, physical examination, or fetal autopsy. For liveborn infants, we collected information on GA at birth, birth weight, Apgar score, localization of lesions, imaging features, and final diagnosis, as well as data on postnatal management. Postnatal diagnosis was separately compared to intrauterine imaging findings, so a retrospective diagnostic test accuracy study was conducted.

### Study protocol of the prenatal imaging

Transabdominal fetal ultrasound (US) examinations were performed using the GE ultrasound device (GE Medical Systems, Kretztechnik GmbH, Zipf, Austria). Standard Doppler ultrasounds of the cervical masses were performed by experienced ultrasound physicians. Ultrasonographic characteristics (including size, location, echogenicity or signal feature, septation, vascularity, turbid fluid, and systolic/diastolic (S/D) ratio) at each examination were recorded. MRI examinations were predominantly used to evaluate the masses producing displacement or invasion of the adjacent tissues. All prenatal MRI images were obtained using a 1.5-T unit (GE Signal HDxt; GE Healthcare, Little Chalfont, UK) and an eight-element phased array body coil. Mothers were placed in a supine or left oblique position without sedation. After a localizing gradient echo sequence, we used single shot fast spin echo (SSFSE) T2-weighted imaging or fast-imaging employing steady-state acquisition (FIESTA), and T1-weighted imaging (liver imaging with volume acceleration-flexible, LAVA-Flex). Diffusion-weighted imaging (DWI) was collected when the masses were solid or solid-cystic. Postnatal results were recorded for the purpose of prognostic management and comparison with antenatal diagnosis. Furthermore, gestational ages, imaging characteristics and measurements at each examination were analyzed.

### Statistical analysis

Data were analyzed using the SPSS software package version 21.0 (IBM SPSS Statistics for Windows, Armonk, NY, USA) and GraphPad Prism Version 6.0 (GraphPad Prism Software, USA). The analyses were performed using student t-tests for continuous variables, and Chi-square test for categorical variables. Based on the information obtained during pregnancy and infancy, survival curve plots were drawn using Kaplan–Meier analysis. All of the statistical tests were two-sided, and *p* ≤ 0.05 were considered statistically significant.
